# Amyloid formation of bovine insulin is retarded in moderately acidic pH and by addition of short-chain alcohols

**DOI:** 10.1007/s00249-019-01420-0

**Published:** 2020-01-04

**Authors:** David Bernson, Almedina Mecinovic, Md Tuhin Abed, Fredrik Limé, Per Jageland, Magnus Palmlöf, Elin K. Esbjörner

**Affiliations:** 1 Division of Chemical Biology, Department of Biology and Biological Engineering, Chalmers University of Technology, Kemivägen 10, 412 96 Gothenburg, Sweden; 2 Nouyron Pulp and Performance Chemicals AB, Separation Products, 445 80 Bohus, Sweden

**Keywords:** Insulin, Aggregation, Amyloid, Kinetics, Thioflavin-T, Circular dichroism

## Abstract

Protein aggregation and amyloid formation are associated with multiple human diseases, but are also a problem in protein production. Understanding how aggregation can be modulated is therefore of importance in both medical and industrial contexts. We have used bovine insulin as a model protein to explore how amyloid formation is affected by buffer pH and by the addition of short-chain alcohols. We find that bovine insulin forms amyloid fibrils, albeit with different rates and resulting fibril morphologies, across a wide pH range (2–7). At pH 4.0, bovine insulin displayed relatively low aggregation propensity in combination with high solubility; this condition was therefore chosen as basis for further exploration of how bovine insulin’s native state can be stabilized in the presence of short-chain alcohols that are relevant because of their common use as eluents in industrial-scale chromatography purification. We found that ethanol and isopropanol are efficient modulators of bovine insulin aggregation, providing a three to four times retardation of the aggregation kinetics at 30–35% (vol/vol) concentration; we attribute this to the formation of oligomers, which we detected by AFM. We discuss this effect in terms of reduced solvent polarity and show, by circular dichroism recordings, that a concomitant change in α-helical packing of the insulin monomer occurs in ethanol. Our results extend current knowledge of how insulin aggregates, and may, although bovine insulin serves as a simplistic model, provide insights into how buffers and additives can be fine-tuned in industrial production of proteins in general and pharmaceutical insulin in particular.

**Electronic supplementary material:**

The online version of this article (10.1007/s00249-019-01420-0) contains supplementary material, which is available to authorized users.

## Introduction

Protein aggregation and formation of amyloid fibrils are underlying causes of many common and devastating disorders (Chiti and Dobson 2006), but can also be a significant problem in the biotechnological industry. Protein aggregation can cause trouble in all phases of protein production as well as during storage and administration of protein biopharmaceuticals (Wang 2005; Frokjaer and Otzen 2005). Amyloid fibrils are highly stable protein homopolymers, which can be formed by most polypeptide chains (Dobson 2001; Fändrich and Dobson 2002). Despite the large structure and size variations among amyloid-forming proteins, the fibrils themselves share important structural and morphological traits; they have diameters around 10 nm, can extend several microns in length, and are typically twisted and unbranched (Sunde et al. 1997). They also share a common cross-β fold and are stabilized by extensive intermolecular hydrogen bonding involving the polypeptide backbone (Sunde et al. 1997). Proteins differ, however, substantially in the propensity and conditions in which they form fibrils. Understanding these characteristics and the effect of extrinsic modulators is important not only in cases where amyloid-forming proteins give rise to disease, but also for the optimization of industrial procedures to avoid or reduce adverse amyloid formation.

Bovine insulin is a 5.8 kDa peptide hormone with 51 amino acid residues, distributed over two polypeptide chains (A and B) that are interconnected by disulphide bridges (Hua et al. 2002). It is produced in β-cells of the pancreatic islets and secreted in response to elevated glucose levels in the blood. In this capacity, the human counterpart has become the most commonly used drug for treatment of diabetes mellitus, and hence one of the world’s major biopharmaceutical products (Walsh 2005). Natively folded insulin, both bovine and human, is predominately α-helical; its three-dimensional structure was solved by X-ray crystallography already in 1969 (Adams et al. (1969)). Insulin functionality is dependent on its quaternary structure; the folded protein can assemble into different oligomers, with the monomer as the biologically active form (Hefford et al. 1986). In aqueous solution, natively folded insulin monomers exist in equilibrium with dimers, tetramers, and hexamers (Brange and Langkjoer 1993; Nettleton et al. 2000; Pekar and Frank 1972). The assembly state is dependent on solution conditions; the monomer is for example favoured at low pH, particularly in acetic acid (Attri et al. 2010), whereas dimers are favoured in hydrochloric acid, and hexamers are strongly stabilized by higher pH and presence of zinc ions (Brange and Langkjoer 1993; Pekar and Frank 1972). Insulin is, in addition, an amyloidogenic protein that readily converts into fibrils in vitro (Waugh 1946); Burke et al. 1972). High concentration, or conditions that destabilize the native state, e.g. low pH and elevated temperatures promote such reactions (Waugh 1946; Brange et al. 1997). Most evidence suggests that insulin fibrillation proceeds from the monomeric state, and that dimer, tetramer, and hexamer formation thereby act as protective in regard to fibril formation (Brange et al. 1997; Ziaunys et al. 2018; Brunetti and Waldhäusl 1987; Nielsen et al. 2001; Waugh et al. 1953). Insulin fibrillation can cause significant complications in diabetes therapy, either directly due to injection site amyloidosis (Nilsson 2016; Dische et al. 1988; Albert et al. 2007; Yumlu et al. 2009), or indirectly by decreasing the long-term stability of insulin formulations (Frokjaer and Otzen 2005; Brange and Langkjoer 1993). It is also a significant problem in industrial-scale production, where insulin aggregation leads to costly problems with column clogging during reverse-phase high-performance liquid chromatography purification (Gusarov et al. 2007; Majors 2004). It is therefore important to understand the fibrillation of this protein, both to increase our understanding of insulin-related amyloidosis, and to improve on existing industrial production procedures.

In this work, we have used biophysical methods to study the amyloid formation of bovine insulin, a common model protein in aggregation studies (Richter and Neises 2005). We have explored how solution pH and short-chain alcohols (common additives and eluents in industrial purification of proteins in general and pharmaceutical human insulin in particular) affect fibril formation rate and fibril morphology, as well as the solubility and structural stability of the native bovine insulin form. The study focuses on the intermediate pH range (pH 4.0) and thus complements and extends the significant body of published data that describe insulin amyloid formation at strongly acidic conditions (pH ≤ 2) (Waugh 1946; Burke et al. 1972; Nielsen et al. 2001).

## Materials and methods

### Materials

Bovine insulin, thioflavin-T (ThT), all buffer chemicals and all solvents were purchased from Sigma-Aldrich. Solvents were of chromatography grade. Bovine insulin was purchased in powdered form (Product No I5500). Thioflavin-T (ThT) was first dissolved in tetrahydrofuran and re-crystallized to remove impurities and then dissolved in distilled water from a milli-Q water system (Merck Millipore, Darmstadt, Germany). The solution was filtered through a 0.2 µm syringe filter to remove undissolved particulates. The ThT concentration was determined by absorption on a Cary 50 absorption spectrophotometer (Agilent Technologies, Santa Clara, California, US), using an extinction coefficient of 36,000 M^−1^ cm^−1^ at 412 nm. Insulin stock solutions were prepared by dissolving powdered bovine insulin in a small amount of 100 mM HCl to ~ 15 mg/mL, followed by dilution into one of the following buffers: 100 mM glycine–HCl, pH 2.2; citrate–phosphate buffer prepared from 100 mM citrate and 200 mM Na_2_HPO_4_ (McIlvaine 1921), spanning from pH 3.0 to 7.0, or 200 mM ammonium acetate, pH 4.0. The insulin concentration was determined by absorption, using an extinction coefficient of *ε*_280_ = 5,800 M^−1^ cm^−1^.

### Aggregation kinetics experiments

Aggregation kinetics was monitored by ThT fluorescence in a Fluostar Omega or Fluostar Optima fluorescence plate reader (BMG Labtech, Ortenberg, Germany) at 60 °C without shaking. Samples were deposited in 100 μl triplicate in black half-area 96-well microtiter plates with transparent bottoms (Corning #3881), and the plates were sealed with plastic film (Bio-Rad Laboratories, Hercules, CA, U.S.) to avoid sample evaporation. ThT fluorescence emission was detected using bottom optics, a 440 ± 10 nm band pass excitation filter, and a 485 ± 12 nm band pass emission filter. The ThT concentration was 7.2 μM in all experiments.

### Solubility and fibril formation yields of insulin

The solubility of insulin as function of pH was estimated by preparing insulin solutions with varying pH at a concentration of 1 mg/ml. The solutions were centrifuged at 13,400 rpm in an Eppendorf Minispin tabletop centrifuge (Eppendorf, Hamburg, Germany) for 15 min to pellet non-dissolved insulin. The solubility was estimated by measuring the insulin concentration in the supernatant by absorption (see above). The same strategy was used to determine fibril formation yields at different pH values, using pre-formed fibrils as starting material.

### Circular dichroism spectroscopy

Circular dichroism (CD) spectroscopy was used to determine the secondary structure of insulin in the presence of ethanol. CD spectra were recorded in 1 nm increments between 190 and 250 nm on a Chirascan spectropolarimeter (Applied Photophysics Ltd, Leatherhead, UK) using quartz cuvettes with 1 mm path length (Hellma GmbH, Mullheim, Germany). The bandwidth was 1 nm, and the integration time was 500 ms. The spectra were corrected for background contributions by subtracting the appropriate buffer blanks.

### AFM and fluorescence imaging

For atomic force microscopy, fibrillar insulin was deposited on freshly cleaved mica and left to settle for 5 min, after which the mica plates were rinsed with milli-Q water and dried under a gentle stream of nitrogen. AFM images were recorded on an NTEGRA Prima setup (NT-MDT, Moscow, Russian Federation) equipped with a gold-coated single crystal silicon cantilever (NSG-01, spring constant ~ 5.1 N/m, resonance frequency of ~ 150 kHz). The images were processed in the Gwyddion software package (Nečas and Klapetek 2012) using planar subtraction, polynomial background subtraction and correction for linear aberrations. For fluorescence microscopy, 150 µl of insulin solution was placed on positively charged glass (Thermo Scientific, Waltham, MA, USA) and sealed with a 18 × 18 mm coverslip. A Zeiss AxioObserver.Z1 inverted fluorescence microscope (Carl Zeiss AG, Oberkochen, Germany) with a 100 × oil immersion objective (NA = 1.46) was used in combination with a Photometrics Evolve EMCCD camera (Photometrics, Tuscon, AZ, USA), 100 ms exposure time, to obtain the images of the samples.

## Results and discussion

### Kinetic assay for insulin amyloid formation, reproducibility and monomer concentration dependence at pH 2.2 and pH 7.0

**Fig. 1:**
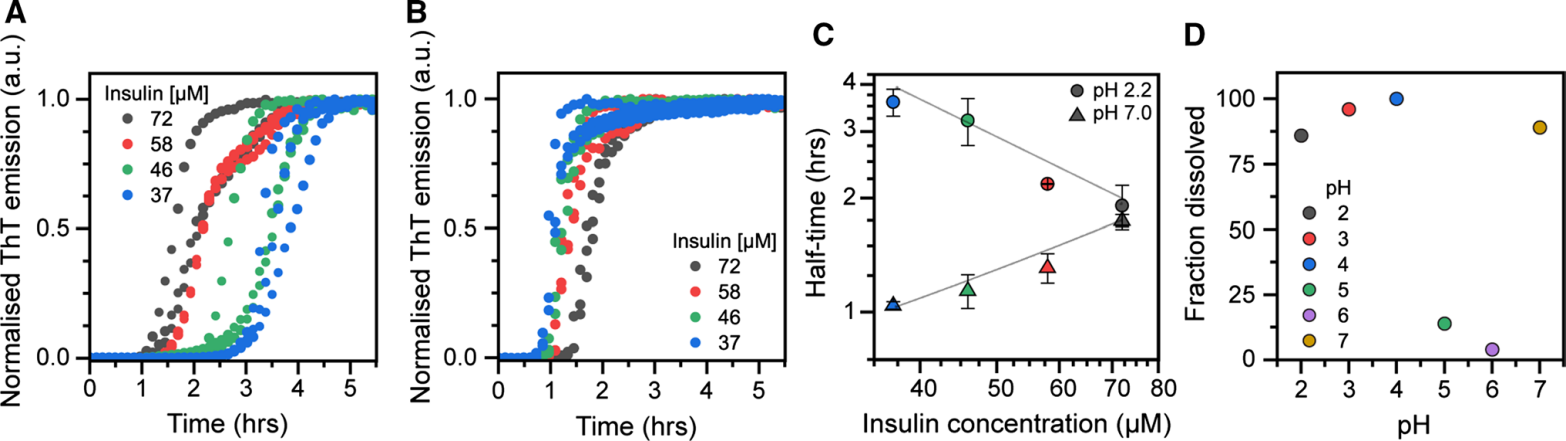
Kinetics of insulin amyloid formation at pH 2.2 and pH 7.0. (A–B) Normalized thioflavin-T (ThT) fluorescence as function of time for insulin solutions with increasing concentration recorded at **a** pH 2.2 and **b** pH 7.0. All experiments were performed in triplicate. **c** Half-time of amyloid formation plotted as function of monomer concentration on a log–log scale. The solid lines indicate linear fits to data with scaling component (slope) of γ =  − 1.02 for pH 2.2 data and γ =  + 0.75 for pH 7.0 data. **d** Percentage of dissolved insulin measured following sedimentation of the insoluble fraction on freshly prepared samples with a total insulin concentration of 172 μM. The insulin was pre-dissolved in 100 mM HCl prior to dilution to the indicated pH as described in Materials and Methods

We set up thioflavin-T kinetic assays to monitor the aggregation of bovine insulin in microtitre plates at 60 °C and under quiescent conditions, initially at pH 2.2 to establish a reproducible method, then extending to pH 7.0 to reflect native conditions. The resulting kinetic curves (presented as normalized ThT fluorescence) are presented in Fig. 1a, b; each condition was analysed in triplicate and all curves are shown. The aggregation rate of bovine insulin increases with increase in monomer concentration at pH 2.2 (Fig. 1a), which is intuitively expected and also consistent with many previous studies (see for example Brange and Langkjoer 1993; Nielsen et al. 2001). Figure 1c shows the reaction half-times, i.e. the time at which 50% of the maximum ThT fluorescence has been reached, as function of the initial insulin concentration on a log–log scale. The scaling component, i.e. the slope of the fitted straight line to the half-time data, for insulin aggregation kinetics at pH 2.2 is  − 1.02, which is consistent with a nucleation-dependent aggregation mechanism (Meisl et al. 2016). At pH 7.0 (Fig. 1b), insulin amyloid formation instead displays an inverse dependence on monomer concentration; the aggregation rate decreases with increase in protein concentration, which is further illustrated by the positive slope (γ =   +   0.75) in the corresponding half-time plot in Fig. 1c. This is, from an amyloid formation point of view, an anomalous behaviour that can be explained by that insulin, in addition to existing as a monomer, populates higher-order oligomeric states that are resilient to amyloid formation; or interfere with fibril elongation (Ziaunys et al. 2018). These higher-order states can be significant, or even predominant, at pH 7.0 despite that no stabilizing zinc was added (Ziaunys et al. 2018; Waugh et al. 1953; Goldman and Carpenter, 1974).

The fibril yields for the reactions shown in Fig. 1 were estimated by centrifugation experiments to sediment amyloid fibrils formed at  a starting concentration of 58 μM insulin. The yield was to 91 ± 8% at pH 2.2 and 90 ± 5% (*n* = 3) at pH 7.0, suggesting that as monomeric insulin is incorporated into fibrils, the equilibrium between monomers and higher-order folded oligomers at pH 7.0 is shifted, such that, e.g. hexamers eventually dissociate, and the resulting monomers thereafter are incorporated into the growing fibrils. The inverse concentration dependence suggests that it is this conversion that is rate limiting in the amyloid forming reaction at pH 7.0. We also used centrifugation to sediment insoluble insulin in 1 mg/ml solutions to investigate the solubility across a range of buffers with different pH values (Fig. 1d). We show that insulin solubility is high across most of the assayed pH range, with the exception of pH 5.0–6.0, which is close to to insulin’s isolectric point (pI = 5.3 (Wintersteiner and Abramson 1933)).

### The fibril formation rate of insulin is the lowest close to the isoelectric point

**Fig. 2:**
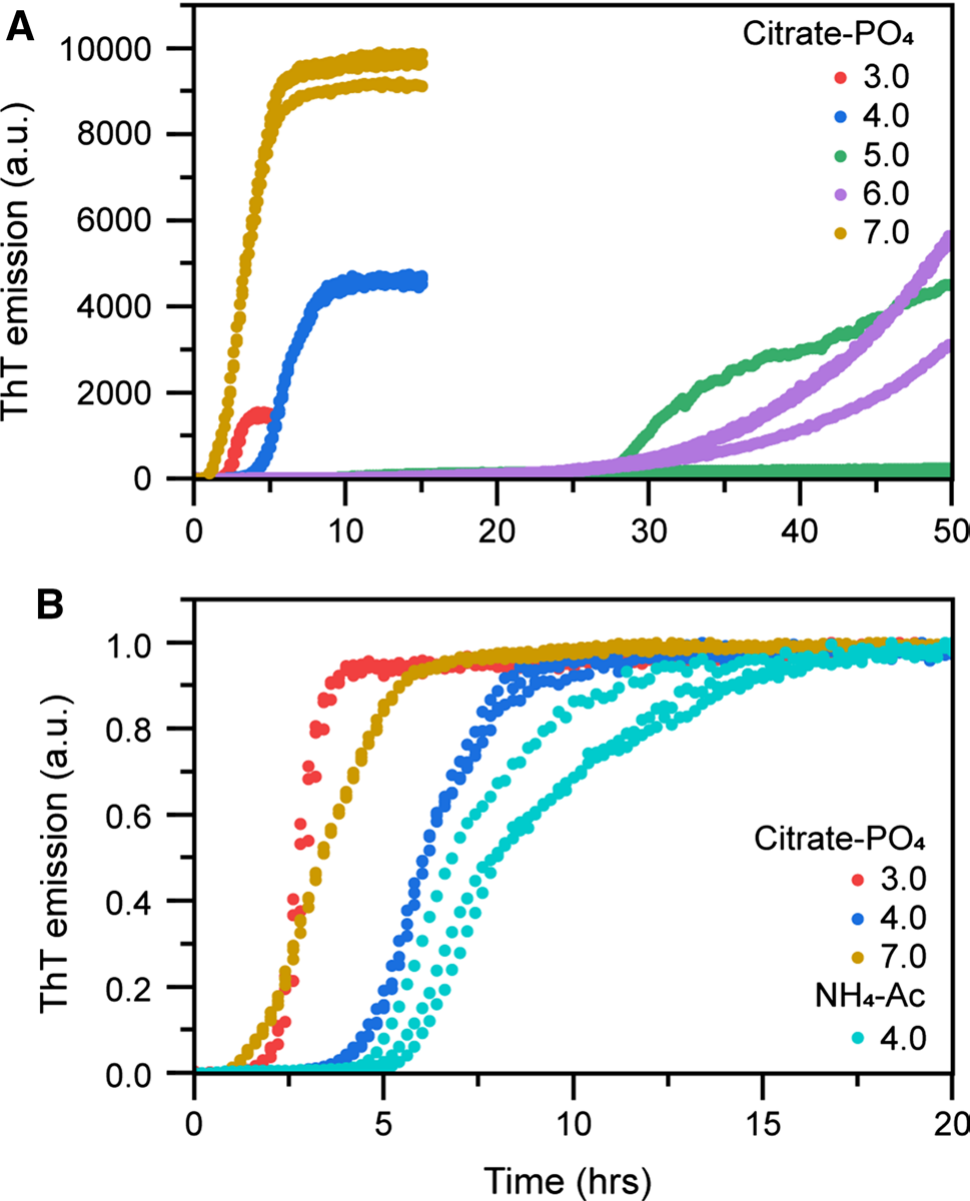
Insulin fibrillation at different pH values. **a** Kinetics of insulin amyloid formation monitored by ThT fluorescence in citrate–phosphate buffers with indicated pH. The initial insulin monomer concentration was 64 μM. **b** Normalized ThT emission from (**a**), including 64 µM insulin aggregated in ammonium acetate buffer with indicated pH. All experiments were performed in triplicate, all replicates are shown in the plot

Next, we monitored the amyloid fibril formation kinetics of bovine insulin at one fixed concentration (64 μM) across a range of different buffer pH values (3.0–7.0). Figure 2a shows ThT fluorescence as function of time. The kinetics of insulin amyloid formation is clearly fastest at pH 3.0 or at pH 7.0 and occurs considerably slower (~ 50% as measured by half-time of fibril formation) at pH 4.0. At intermediate pH (5.0–6.0), close to insulin’s isoelectric point, amyloid formation is strongly inhibited, thereby hindering measurement of reliable half-times within the assayed time range. The aggregation rate is not only slowed down, but the reactions also become more stochastic in the intermediate pH range. This is particularly apparent at pH 5.0, which is very near insulin’s isoelectric point; charge neutralization appears in this case to impede rather than to accelerate self-assembly. In this respect, insulin behaves differently from for example the Alzheimer’s disease-related amyloid-β peptide or the Parkinson protein α-synuclein where the reduced electrostatic repulsion close to their respective pI act to catalyse their fibrillation (Guo et al. 2005). Furthermore, the slow aggregation as well as apparently stochastic kinetics that insulin displays at pH 5.0–6.0 coincides with low solubility of the protein (Fig. 1d) and supports that near insulin’s pI, amyloid formation occurs in competition with precipitation.

**Fig. 3:**
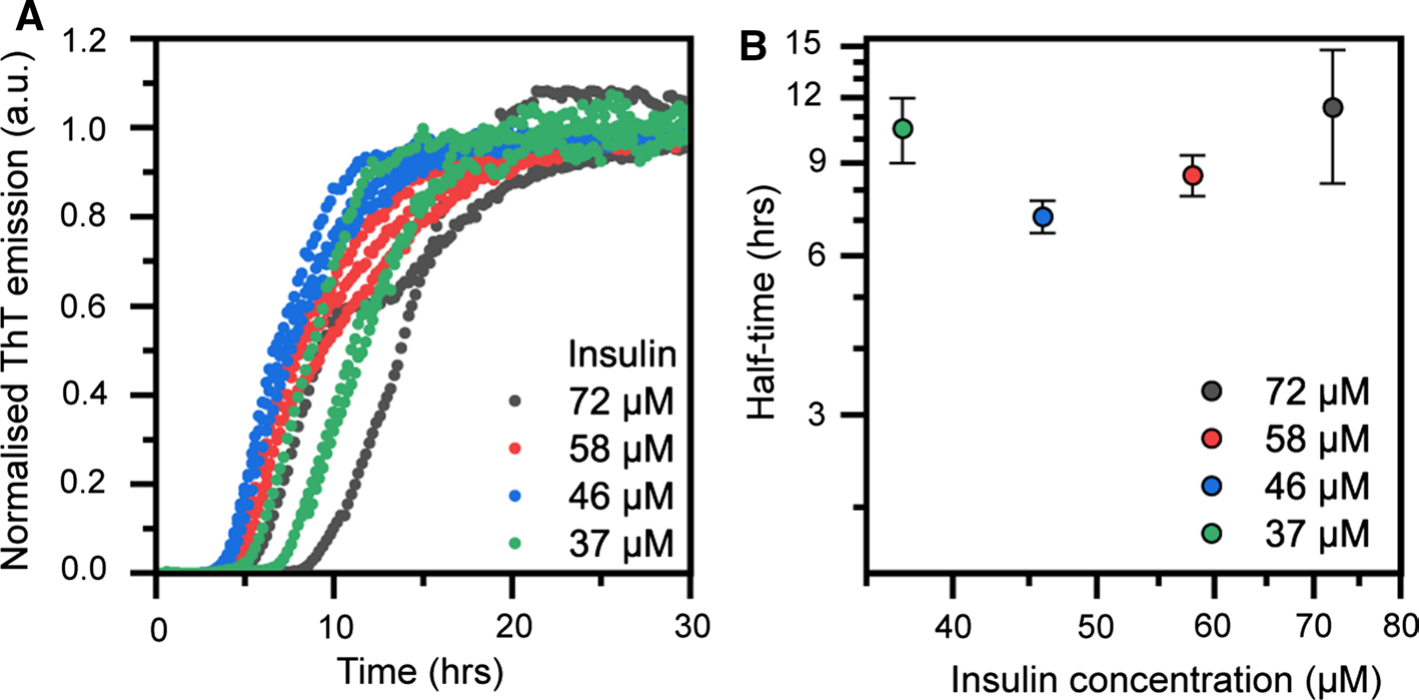
(**a**) Kinetics of insulin amyloid formation monitored by ThT fluorescence in ammonium acetate, pH 4.0, at different insulin concentrations. **b** Reaction half-times extracted from the kinetic traces in (**a**) as function of insulin concentration. Error bars represent standard deviation (*n* = 3)

By comparing the aggregation reaction rates in Fig. 2a with measurements of insulin solubility (Fig. 1d), we note that at pH 4.0, insulin amyloid formation is retarded, but the solubility remains high. From a protein purification perspective, this is clearly an advantageous situation; conspicuously, these also appear to be commonly used conditions in pharmaceutical production of the human insulin form. In industrial settings, ammonium acetate buffer is often used for purification at pH 4.0. We therefore compared the aggregation rate in citrate–phosphate and ammonium phosphate (Fig. 2b), showing a somewhat slower rate in the latter (half-time of 7.7 ± 0.8 h compared to 6.5 ± 0.1 h in citrate phosphate). We foremost attribute this to the differences in ionic strength of the two buffer systems, as salt concentration has been demonstrated to be important for insulin’s aggregation rate (Nielsen et al. 2001). We also examined the concentration dependence of insulin fibrillation in ammonium acetate, pH 4.0 (Fig. 3). It is clear both from the aggregation curves (Fig. 3a) and half-times (Fig. 3b) that at this intermediate pH, insulin does not have a clear concentration-dependent aggregation behaviour. It thus behaves differently, and in some sense intermediate, from what is seen at pH 2.2 and pH 7.0.

**Fig. 4:**
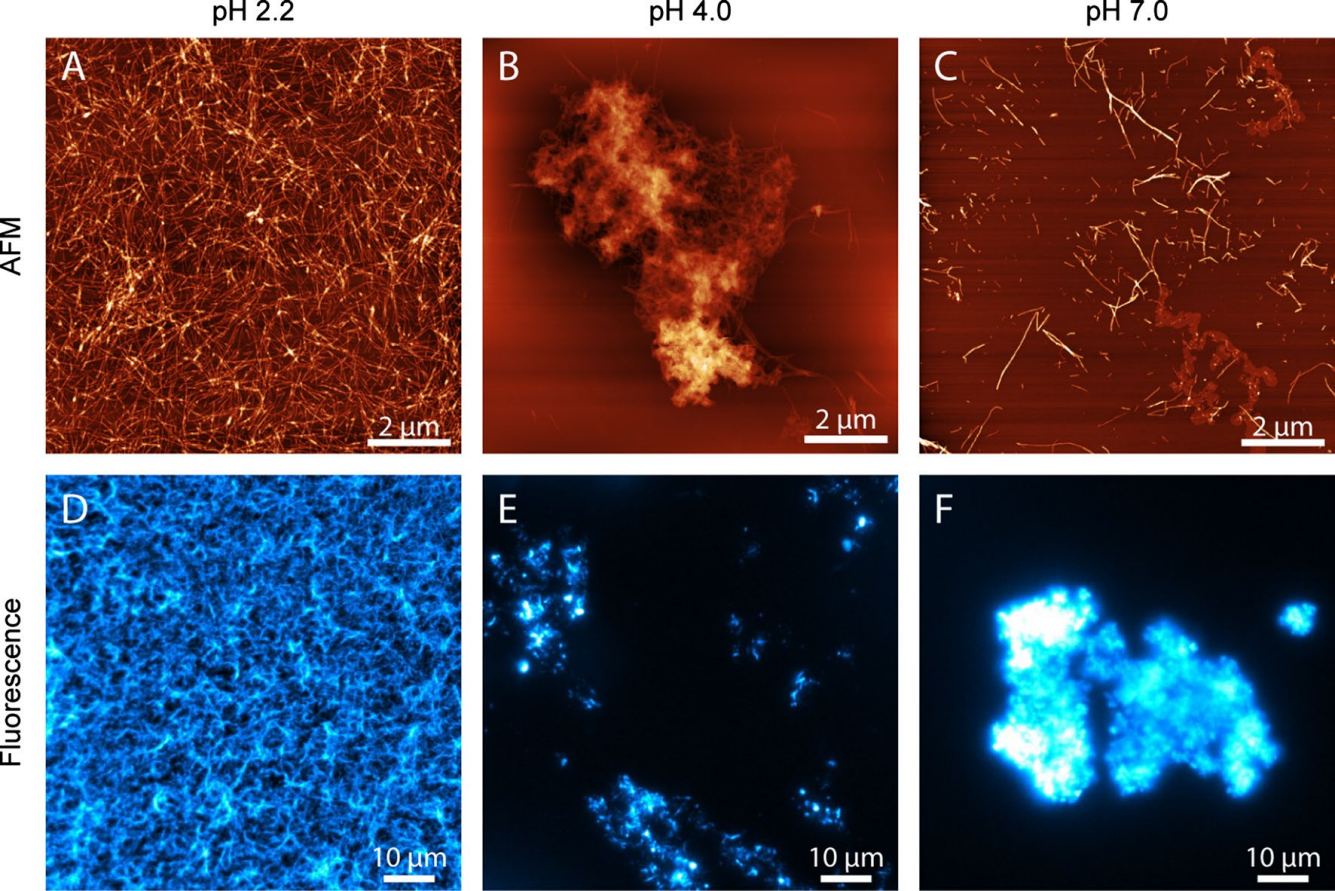
Insulin fibrils aggregated at pH 2.2 (left), pH 4 (middle) and pH 7 (right), imaged by atomic force microscopy (**a**–**c**) or ThT fluorescence microscopy (**d**–**f**). The scale bar is 2 µm in the AFM images, and 10 µm in the fluorescence images

Next, we examined the morphological appearance of insulin fibrils formed at pH 2.2, pH 4.0 and pH 7.0 using both atomic force microscopy (AFM), and fluorescence imaging with ThT as counterstain (Fig. 4). The images confirm that the ThT fluorescence that we observe in the kinetic experiments is consistent with the formation of amyloid fibrils in all cases, also at pH 4.0, which is reasonably close to the isoelectric point and thus conditions where native insulin becomes insoluble. The morphology of the ensuing insulin fibrils differs, however, distinctly with pH. At pH 2.2, aggregated insulin deposits on mica as individual fibrils and at a high concentration; they were also always evenly dispersed over the entire mica substrate. This agrees very well with the corresponding fluorescence images (which were acquired on fibrils adsorbed on a glass substrate in aqueous solution). At pH 4.0 and 7.0, the fibrils behave differently. First, they were considerably more difficult to locate and image on the mica substrate, despite that we found the fibril yields at the different pH values to be high in all cases (for pH 4.0, the fibril yield was > 99.5%). At pH 4.0 and pH 7.0, the insulin amyloid fibrils tend to form dense clusters in addition to dispersed fibrils. This is supported by the fluorescence images that show very bright macroscopic aggregates in the field of view. These structures were in fact so brightly fluorescent compared to the fibrils imaged at pH 2.2 that we had to bleach the fluorescence before recording the actual images to avoid oversaturating the detector.

**Fig. 5:**
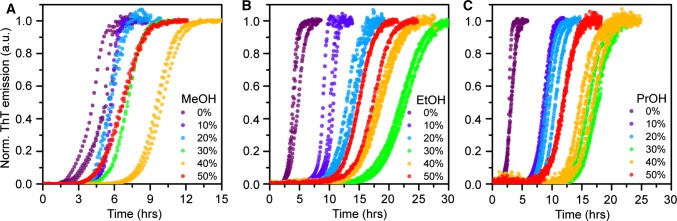
Effect of alcohols on insulin fibrillation kinetics at pH 4.0. Amyloid formation kinetics monitored by ThT fluorescence in ammonium acetate buffer with increasing concentrations of **a** methanol **b** ethanol, and **c** isopropanol. All kinetic data were recorded in triplicate and all traces are shown in the figures. The percentage of solvent refers to volume fraction

To mimic conditions relevant to industrial purification of insulin, we investigated the effect of three short-chain alcohols (methanol, ethanol and isopropanol) on insulin aggregation in ammonium acetate buffer. We monitored ThT kinetics as function of increasing concentration (range: 0–50% v/v) of each alcohol (Fig. 5a–c). All three alcohols initially retarded fibril formation in a concentration-dependent manner up to a certain volume fraction, whereafter the rate was again observed to increase, suggesting that the solvent concentration in eluting buffers can be optimized to obtain maximum elution and, simultaneously, reduce the risk of aggregation. This behaviour is also depicted by the change in half-time as function of alcohol concentration shown in Fig. 5a. Ethanol and isopropanol can retard fibril formation at pH 4.0 by as much as three to four times at the most effective alcohol:buffer mixing ratio, which according to our result is around 30–35%. Methanol is apparently a weaker modulator (it retards fibril formation by  ~ 2 times); higher concentrations (40%) are also needed to reach maximal effect. The finding that methanol is less efficient than ethanol and isopropanol suggests that fibril formation of bovine insulin is significantly dependent on solvent polarity, with mixing in of increasingly non-polar additives having a retarding (aggregation-protective) effect.

**Fig. 6:**
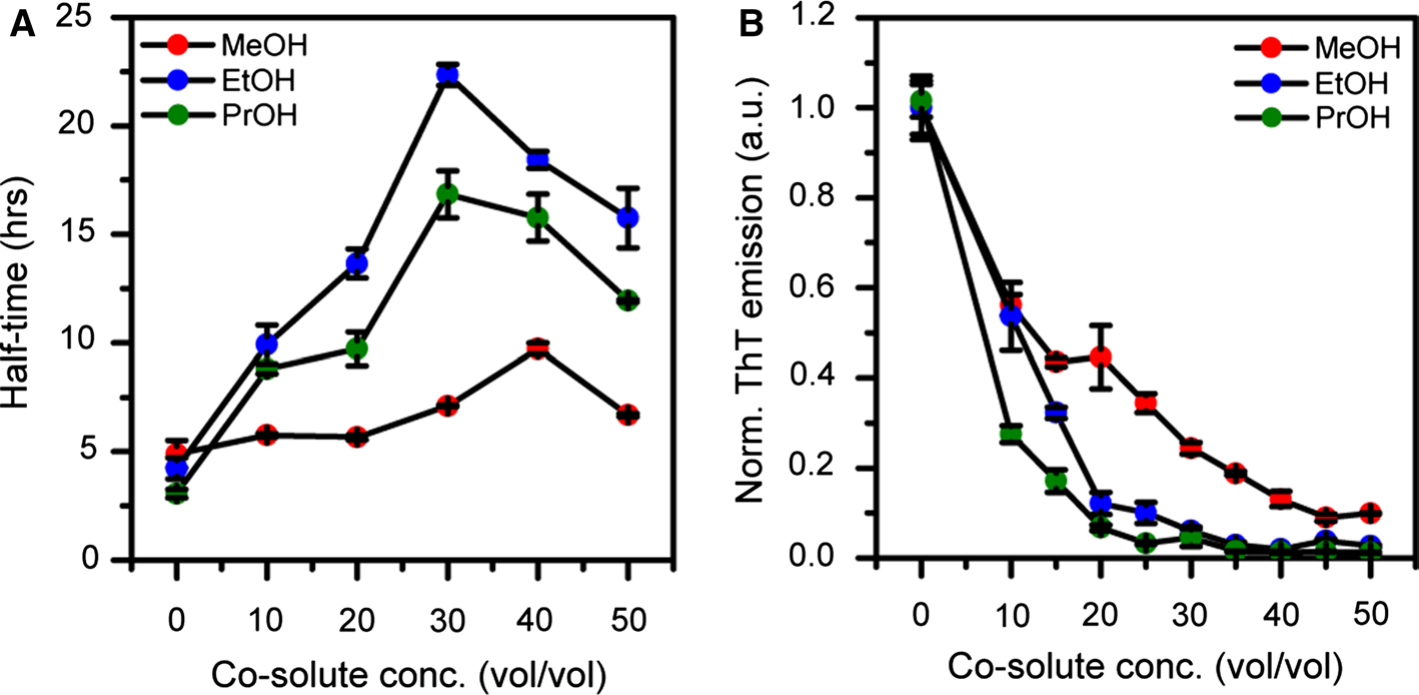
**a** Reaction half-times extracted from the data in Fig. 4 for the kinetics of insulin amyloid formation in ammonium acetate buffer containing increasing volume fractions of respectively methanol (red, MeOH), ethanol (blue, EtOH), and isopropanol (green, PrOH). **b** Change in end-point ThT fluorescence for the kinetic traces shown in Fig. 4 reported as function of alcohol concentration for methanol (red), ethanol (blue), and isopropanol (green). The error bars represent standard deviation (*n* = 3)

**Fig. 7:**
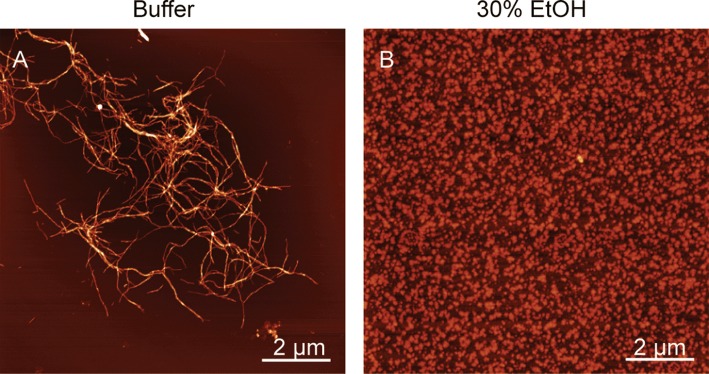
(**a**–**b**) Atomic force microscopy imaging of insulin aggregated in ammonium acetate, pH 4.0, in the absence (**a**) and presence (**b**) of 30% (vol/vol) EtOH. The scale bar is 2 µm

We also report the change in the end-point ThT fluorescence as function of alcohol concentration; these data were extracted from the non-normalized raw data corresponding to the kinetic curves shown in Fig. 5. The end-point values (Fig. 6b) decrease steadily and significantly with increasing alcohol concentration, but do not correlate with the half-times in Fig. 6a. This is probably due to ThT’s fluorescence being sensitive both to ThT binding affinity and to the quantum yield of each bound dye molecule (Lindberg et al.^2015^, ^2017^). To further investigate the effect of ethanol on insulin during fibrillation, we used AFM to image samples from the end point of the fibrillation reaction. As seen in Fig. 7a, insulin readily forms classic amyloid fibrils in ammonium acetate, pH 4.0, whereas the sample aggregated in the presence of 30% EtOH (Fig. 7b) contains, at high abundance, oligomeric particles,  ~ 2–3 nm in size, which could be consistent with dimers to hexamers (reported as 20 × 40 Å, and 35 × 50 Å, respectively (Blundell et al. 1972)). Our data thus suggest that ethanol as co-solvent drives insulin into oligomeric assemblies, which are likely off-pathway due to the reduction in aggregation rate.

**Fig. 8:**
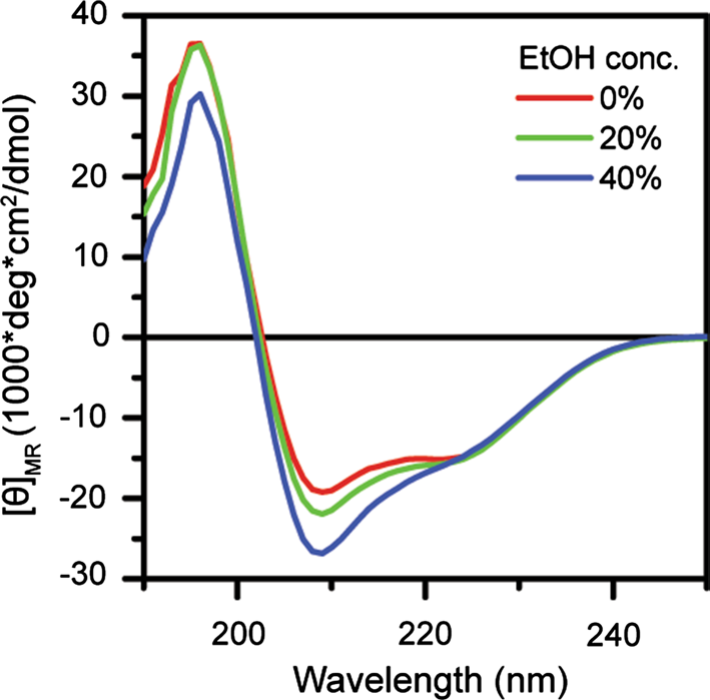
Far-UV CD spectra showing conformational change of insulin induced by ethanol co-solvation at pH 4.0

Lastly, to further explore why alcohols modulate the amyloid formation kinetics of bovine insulin, we used CD spectroscopy to examine the secondary structure of non-aggregated insulin at pH 4.0 in solutions with increasing concentrations of ethanol. The resulting CD spectra in Fig. 8 show that ethanol up to a concentration of 40% acts to enhance the negative ellipticity in the 208 nm absorption band relative to the 222 nm absorption band. This can be attributed to the relative packing of α-helices in the protein native state; specifically, a decrease in the 222/208 nm ratio is indicative of unpacking of coiled helices (Lau et al. 1984; McNamara et al. 2008). Similar behaviour has been reported previously in relation to destabilization of the insulin dimer by ethanol at low pH and could thus explain the increase in amyloid fibril formation rate ≥ 30% ethanol (vol/vol) (Brems et al. 1990; Dzwolak et al. 2005).

## Conclusions

We have studied the amyloid formation kinetics of insulin as a function of protein concentration and pH and in the presence of increasing concentrations of short-chain alcohols at pH 4.0, a pH that we identified as particularly interesting due to its common use in reverse-phase HPLC purification of pharmaceutical human insulin and because of our findings that it presents the best combination of high solubility and slow aggregation rate. This is advantageous and important from the perspective of avoiding adverse aggregation in the industrial manufacturing process. Further, by examining the concentration dependence on insulin aggregation at pH 2.2, we confirm previous observations suggesting that the aggregation mechanism is dependent not only on primary nucleation, but strongly and decisively on secondary and auto-catalytic processes. At pH 7.0 we find that the aggregation behaviour is modulated by the ability of the insulin monomer to associate into natively folded oligomers, presumably hexamers. Therefore, conditions that monomerize insulin at physiological pH also render the protein more susceptible to aggregation. Finally, we demonstrate that at pH 4.0, folded insulin can be stabilized by alcohols, resulting in significant retardation of aggregation rates, presumably due to the formation of off-pathway oligomers. We find that ethanol and isopropanol are better stabilizers than methanol, and that these two alcohols increase the reaction half-times nearly linearly up to a concentration of 35–40% (volume fraction). Altogether, our study provides details of insulin fibrillation kinetics in different solution conditions relevant to pharmaceutical production. It shows that pH 4.0 provides optimal, aggregation-limited conditions which are well suited for purification, and suggests that further stabilization of the native insulin can be obtained by common solvents used as eluents by the pharmaceutical industry.

## Electronic supplementary material

Below is the link to the electronic supplementary material.

Supplementary file1 (PDF 54 kb)

## Abbreviations

ThT: Thioflavin-T
CD: Circular dichroism
AFM: Atomic force microscopy
