# Supplementary material for: Amyloid formation of bovine insulin is retarded in moderately acidic pH and by addition of short-chain alcohols
Source: Eur Biophys J. 2020 Jan 4;49(2):145–53. doi: 10.1007/s00249-019-01420-0 (PMC7069927; doi:10.1007/s00249-019-01420-0)
Supplement: Supplementary file 1 — Supplementary file1 (PDF 54 kb) [file 249_2019_1420_MOESM1_ESM.pdf]

Supporting Information to:

Amyloid formation of bovine insulin is retarded in moderately acidic pH and by addition of short-chain alcohols

David Bernson[1,§], Almedina Mecinovic[1,§], Md Tuhin Abed[1], Fredrik Limé[2], Per Jageland[2], Magnus Palmlöf[2], Elin K. Esbjörner[1,*]

[1]Division of Chemical Biology, Department of Biology and Biological Engineering, Chalmers University of Technology, Kemivägen 10, 412 96 Gothenburg, Sweden

[2]Noyron Pulp and Performance Chemicals AB, Separation Products, SE 445 80 Bohus, Sweden

[§]shared first authors

*Corresponding author: email: eline@chalmers.se, phone: +46-31-772 5120; ORCID: 0000-0002-1253-6342

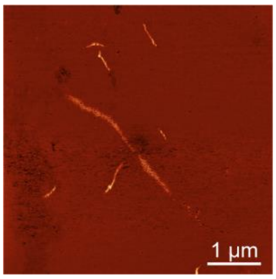

Atomic force microscopy imaging of insulin aggregated in ammonium acetate, pH 4.0 in presence of 30% (vol/vol) EtOH. The sample was diluted 100 times before deposited on the mica surface. Fibrillar species with typical amyloid morphology are present. The scale bar is 1 µm.
